# Updates on the Activity, Efficacy and Emerging Mechanisms of Resistance to Cefiderocol

**DOI:** 10.3390/cimb46120846

**Published:** 2024-12-14

**Authors:** Gabriele Bianco, Matteo Boattini, Monica Cricca, Lucia Diella, Milo Gatti, Luca Rossi, Michele Bartoletti, Vittorio Sambri, Caterina Signoretto, Rossella Fonnesu, Sara Comini, Paolo Gaibani

**Affiliations:** 1 Department of Experimental Medicine, University of Salento, 73100 Lecce, Italy; gabrielebnc87@gmail.com; 2 Department of Public Health and Paediatrics, University of Torino, 10124 Turin, Italy; matteoboattini@gmail.com; 3 Microbiology and Virology Unit, University Hospital Città della Salute e della Scienza di Torino, 10129 Turin, Italy; 4 Lisbon Academic Medical Centre, 1000-001 Lisbon, Portugal; 5 Department of Medical and Surgical Sciences-DIMEC, Alma Mater Studiorum, Section Microbiology, University of Bologna, 40138 Bologna, Italy; monica.cricca3@unibo.it (M.C.); vittorio.sambri@unibo.it (V.S.); 6 Unit of Microbiology, The Great Romagna Hub Laboratory, 47522 Cesena, Italy; 7 Department of Biomedical Sciences, Humanitas University, 20089 Milan, Italy; lucia.diella@humanitas.it (L.D.); michele.bartoletti@hunimed.eu (M.B.); 8 Department of Medical and Surgical Sciences, Alma Mater Studiorum, Section Pharmacology, University of Bologna, 40138 Bologna, Italy; milo.gatti2@unibo.it; 9 Department of Diagnostics and Public Health, Microbiology Section, Verona University, 37134 Verona, Italy; dr.rossiluca@gmail.com (L.R.); caterina.signoretto@univr.it (C.S.); 10 Microbiology and Virology Unit, Azienda Ospedaliera Universitaria Integrata Di Verona, 37134 Verona, Italy; rossella.fonnesu@aovr.veneto.it; 11 Operative Unit of Clinical Pathology, Carlo Urbani Hospital, 60035 Jesi, Italy; comini.sara@gmail.com

**Keywords:** antimicrobials, cefiderocol, in vitro activity, multidrug resistance, Gram-negative, emerging resistance

## Abstract

In recent years, novel antimicrobials have been developed to counter the emergence of antimicrobial resistance and provide effective therapeutic options against multidrug-resistant (MDR) Gram-negative bacilli (GNB). Cefiderocol, a siderophore cephalosporin, represents a novel valuable antimicrobial drug for the treatment of infections caused by MDR-GNB. The mechanism of cefiderocol to penetrate through the outer membrane of bacterial cells, termed “*Trojan horse*”, makes this antimicrobial drug unique and immune to the various resistance strategies adopted by GNB. Its broad spectrum of action, potent antibacterial activity, pharmacokinetics properties, safety, and tolerability make cefiderocol a key drug for the treatment of infections due to MDR strains. Although this novel antimicrobial molecule contributed to revolutionizing the therapeutic armamentarium against MDR-GNB, the recent emergence of cefiderocol-resistant strains has redefined its role in clinical practice and required new strategies to preserve its antibacterial activity. In this review, we provide an updated discussion regarding the mechanism of action, emerging mechanisms of resistance, pharmacokinetic/pharmacodynamic (PK/PD) properties, and efficacy data of cefiderocol against the major Gram-negative bacteria and future prospects.

## 1. Introduction 

Multidrug-resistant (MDR) Gram-negative bacterial infections are among the most significant threats to human health, especially for critically ill and hospitalized patients [1]. In recent years, several therapeutic agents have been developed to enhance the anti-infective arsenal against difficult-to-treat (DTR) Gram-negative bacteria (GNB) [2,3]. However, effective therapies for metallo-β-lactamase (MBL)-producing *Enterobacterales* and glucose-nonfermenting Gram-negative organisms, such as *Pseudomonas aeruginosa*, *Acinetobacter baumannii* complex, and *Stenotrophomonas maltophilia*, remain scarce [4,5].

Cefiderocol was approved in the USA in 2019 and in Europe in 2020 for the treatment of infections caused by Gram-negative microorganisms in adult patients. The cefiderocol prescription is specifically limited to patients who have limited or no alternative treatment options, such as those with infections caused by carbapenem-resistant (CR) *Enterobacterales* (CRE), *P. aeruginosa* (CRPa)*, A. baumannii* (CRAb)*,* and other difficult-to-treat pathogens. As a result, cefiderocol has become a key agent in the armamentarium against CR Gram-negative infections [6]. Cefiderocol is a novel siderophore cephalosporin that employs a “Trojan horse” strategy to enter the bacterial cell; it binds to extracellular free iron and utilizes bacterially active iron transport channels to hijack the cell and penetrate the outer cell membrane [7]. Cefiderocol primarily targets penicillin-binding protein 3 (PBP3), disrupting cell wall synthesis [8]; however, modifications to PBP3 may confer resistance to cefiderocol [9]. 

Cefiderocol circumvents resistance mechanisms related to porin channel mutations and upregulated efflux pumps; it also possesses intrinsic stability against hydrolysis by carbapenemases [7]. In particular, its side-chain properties confer high stability against hydrolysis by a range of β-lactamases, including serine β-lactamases such as extended-spectrum β-lactamase (ESBLs), and MBL. This makes cefiderocol one of the few effective options against difficult-to-treat Gram-negative bacteria that produce MBL, making it particularly useful in treating infections caused by pathogens resistant to almost all other β-lactams [10].

According to large cohort studies [11,12,13], cefiderocol demonstrates effectiveness against most strains of Enterobacterales, *P. aeruginosa, S. maltophilia*, and *A. baumannii*, including those resistant to carbapenems, β-lactam/β-lactamase inhibitor (BL-BLIC) combinations, and polymyxins. However, resistance to cefiderocol has been reported in certain cohorts [14,15,16], and increasing evidence highlights the emergence of resistance during treatment [9,17,18,19,20].

In this review, we summarize the mode of action, the emerging characteristics associated with cefiderocol resistance, and its use in clinical practice considering its pharmacokinetic/pharmacodynamic (PK/PD) targets and its role in real life.

## 2. Mechanism of Action and In Vitro Activity

**Figure 1:**
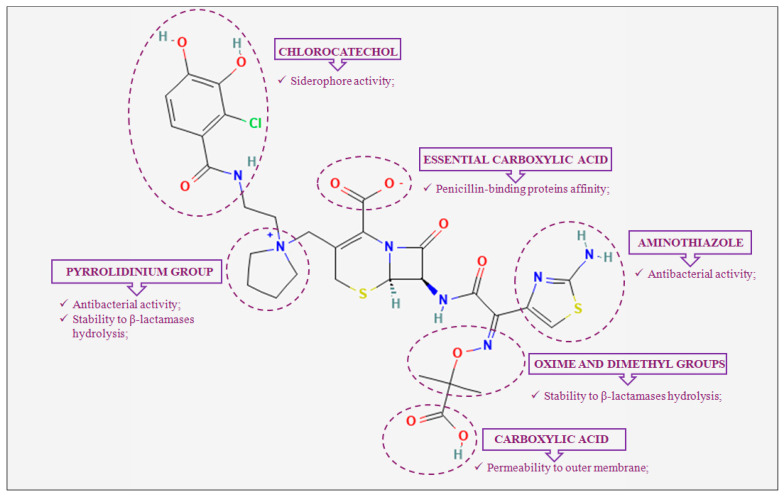
Two-dimensional representation of cefiderocol. Structure activity relationship on Gram-negative bacteria highlighted by the violet dotted circles.

**Figure 2:**
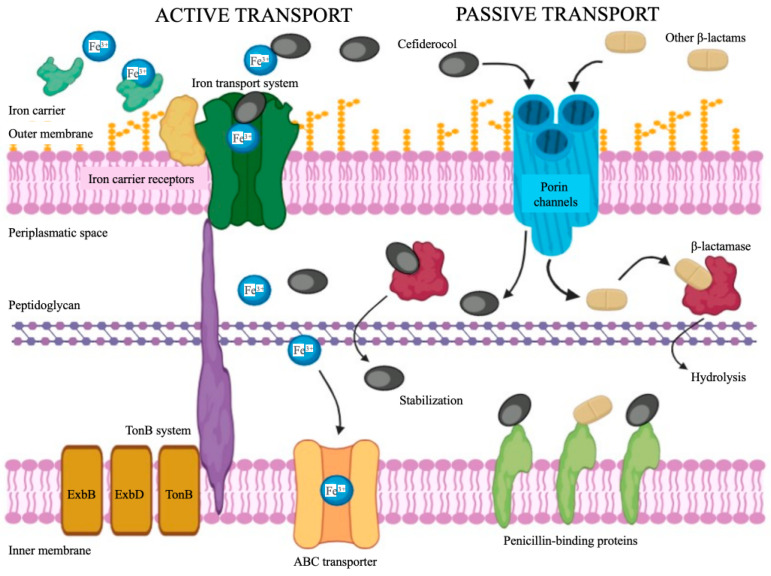
Mechanisms of action of cefiderocol on Gram-negative bacteria.

Cefiderocol (formerly S-649266, GSK2696266) is a new catechol-conjugated cephalosporin developed and marketed by Shionogi & Co., Ltd. (Osaka, Japan) as a promising drug for the treatment of multidrug-resistant Gram-negative bacilli infections [21]. Its structure, which has similar features to those of cefepime and ceftazidime, is characterized by several constituent groups that are the basis of its multiple mechanisms of action (Figure 1) [22]. On the C-7 side chain, aminothiazole, oxime and dimethyl groups, and carboxylic acid provide antibacterial activity, stability to β-lactamases hydrolysis, and permeability to the outer membrane, respectively [22]. On the C-3 side chain, essential carboxylic acid, the pyrrolidinium group, and chlorocathecol improve penicillin-binding protein affinity, antibacterial activity, and stability to β-lactamases hydrolysis, and also confer the ability to bind free ferric ions, respectively [22]. It is precisely this latter siderophore activity in the extracellular environment that represents the unique and distinctive mechanism of action of cefiderocol. In fact, this property, which has been renamed the “Trojan horse” strategy, allows cefiderocol to enter the bacterial cell via the iron transport system, overcoming resistance mechanisms responsible for outer membrane impermeability (i.e., regulation efflux pump, loss of the porin channel) (Figure 2).

The high activity of cefiderocol has been reported in several in vitro studies (Table 1) [23,24,25,26,27,28,29,30,31,32,33,34,35,36,37,38,39,40,41,42,43,44,45,46,47,48,49], and the most robust reported in a recent systematic review and meta-analysis [23].

Karakonstantis et al. analyzed 78 studies reporting more than 82,000 worldwide clinical isolates. The prevalence of cefiderocol non-susceptibility was low overall but varied according to (1) species (Enterobacterales 3.0%, *P. aeruginosa* 1.4%, *A. baumannii* 8.8%, and *S. maltophilia* 0.4%), (2) phenotype (CRE 12.4% and CRAb 13.2%), and (3) genotype (New Delhi MBL-producing Enterobacterales 38.8%, New Delhi MBL-producing *P. aeruginosa* 22.9%, New Delhi MBL-producing A. *baumannii* 44.7%, and ceftazidime/avibactam-resistant Enterobacterales 36.6%) [23]. Further evidence has been added to these data over the past year. Data from the main published series show that cefiderocol retains potent activity (>90%) against the main Gram-negative bacteria, with the exception of K. pneumoniae strains reported in an Indian study [24] and P. *aeruginosa* [33], S. *maltophilia*, Achromobacter, and Burkholderia species isolates [39] detected from patients with cystic fibrosis. The data on susceptibility to cefiderocol tended to be reduced in sub-analyses that only considered collections of carbapenem non-susceptible strains, mainly involving K. *pneumoniae* (80.6%) [24], P. *aeruginosa* (60.3% and 70.6%) [33,36], A. *baumannii* (85%) [35], and Achromobacter species (88.5%) [42]. The suboptimal activity of cefiderocol has also been reported in recent Czech [28] and Turkish [46] studies on carbapenem-non-susceptible Enterobacterales (82%), P. *aeruginosa* (84%), and A. *baumannii* (59.6%) isolates, respectively. Among studies characterizing carbapenemase-producing strains, cefiderocol showed <90% activity against Enterobacterales (86–90%, 83.4–87.5%, 83.3%) [27,40,48] and P. *aeruginosa* (69%, 79.5%) strain collections [34,44]. In all cases, the most common mechanism of resistance is MBL production, especially the New Delhi MBL-type. In contrast, over 90% activity of cefiderocol against carbapenemase-producing A. baumannii (91.2% and 97.9%) has been shown in two reports of Japanese and American bacterial surveillance programs [47,48].

Cefiderocol is a siderophore cephalosporin able to chelate iron ions (Fe^3+^). This property allows it to enter the bacterial periplasm via iron-carrying outer membrane transport protein (TonB-dependent transport protein). This process of using the bacterial iron acquisition system to enhance the outer membrane permeation is known as the “Trojan horse” strategy. The energy required for this process is provided by the membrane potential and mediated by the endosomal energy transduction TonB–ExbB–ExbD protein complex. This dual entry strategy promotes a faster and higher concentration of the antibiotic in the periplasm. Moreover, the chemical structure of cefiderocol renders this antibiotic more stable against a variety of Ambler A, B, C, and D β-lactamases that are concentrated in the periplasmatic space.

## 3. Mechanisms of Cefiderocol Resistance

The key characteristics of cefiderocol are its active uptake mechanisms by Gram-negative bacteria under iron-depleted conditions and its improved stability to the hydrolytic activity of various types of β-lactamases. However, several mechanisms may be involved in the acquisition of cefiderocol resistance, and they can be summarized into four groups: mutations in genes related to iron transfer systems, the expression of β-lactamases, mutations in penicillin-binding proteins, porin loss, or efflux pump overexpression. Each of these mechanisms alone is generally not sufficient to increase cefiderocol MICs above PK/PD breakpoints. Therefore, resistance to cefiderocol is typically acquired as a consequence of various combinations of the above resistance mechanisms (Table 2).

### 3.1. Mutations in Genes Related to Iron Transfer Systems

The role of siderophore receptor mutations in cefiderocol resistance has been shown by studies both on isogenic mutants and clinical isolates [9]. Among the receptors recognized for cefiderocol uptake in *P. aeruginosa* and *A. baumannii* are two *ton*B-dependent receptors (TBDRs), PiuA and PirA [50]. Inactivation of *piuA* has been associated with a 16 to 32-fold increase of cefiderocol MIC in *P. aeruginosa* mutants [50,51]. Similarly, the deletion of *piuD* (an ortholog of piuA) increased cefiderocol MICs 32-fold in *P. aeruginosa* [51]. Moreover, the deletion of *pirA* in *P. aeruginosa* was associated with low (two-fold increase) or no impact on cefiderocol MIC [50,51], but combining the deletion of *piuA* or *piuD* with the deletion of *pirA*, the increase was 32-fold and 64-fold, respectively [50,51]. The in vivo emergence of cefiderocol resistance involving iron transport receptors has been reported in patients treated with ceftazidime/avibactam or ceftolozane/tazobactam [52,53]. Gomis-Font et al. reported the emergence of in vivo cefiderocol resistance development in *P. aeruginosa* caused by a large genomic deletion, including the *piuDC* region and the ampC regulator ampD, in the absence of cefiderocol treatment. This deletion led to co-resistance to cefiderocol and ceftazidime/avibactam as a result of ampC overexpression and PiuDC loss [52]. Streling et al. reported a *P. aeruginosa* isolate that acquired resistance to cefiderocol after ceftolozane/tazobactam treatment and exhibited the substitution L147F in the ampC gene together with mutations in *piuA* and *pirR* genes compared to the ancestor isolate [53]. The relevant role of *piuA* in cefiderocol resistance has also been demonstrated in *A. baumannii*. The genomic analysis of cefiderocol-resistant *A. baumannii* clinical isolates revealed the deficiency of *piuA* alone or associated with reduced expression of *pirA* [54,55]. Recently, Findlay et al. reported the in vivo emergence of cefiderocol resistance following 10 days of cefiderocol treatment in a burn ICU patient with *A. baumannii* bloodstream infection. The WGS analysis of cefiderocol-resistant and the ancestor cefiderocol-susceptible isolate revealed three nucleotide deletions upstream of *piuA* causing the down expression of the gene [56]. Huang et al. identified *pirA* inactivation of by insertion of an ISAba36 element as a key resistance mechanism following cefiderocol treatment in a patient with OXA-72-producing *A. baumannii* infection [57].

In Enterobacterales, alterations in siderophore receptors c*irA* and/or *fiu* have been associated with reduced susceptibility or resistance to cefiderocol. Various mutations in *cirA* resulted in increased cefiderocol MIC values in *K. pneumoniae* and *E. cloacae* complex strains, both in isogenic mutants and in vitro-induced resistant strains [58,59]. Most of the cefiderocol-resistant *K. pneumoniae* or *E. coli* clinical isolates presenting mutations in cirA were also producers of NDM MBL, highlighting the role of certain β-lactamases in contributing to resistance [63,64,65,66,67]. Experiments on isogenic mutants of *E. coli* showed that the loss of *fiu* resulted in a two-fold increase of cefiderocol MIC, increasing up to 16-fold when combined with deletion of cirA [50]. Mutations in other iron uptake and transport-related genes (e.g., f*huA, fepA, iutA, cirA, sitC, apbC, fepG, fepC, fetB, yicI, yicJ*, and *yicL*) were identified in cefiderocol-resistant *K. pneumoniae* isolates [95]. Alterations in genes encoding three inner membrane proteins, which transfer energy to the outer membrane for iron transport (TonB–ExbB–ExbD); other tonB-dependent receptors (*fecA*); a ferric iron ABC transporter (*fbpA*); and an iron uptake system component (*efeo*) were also identified in in vitro-derived cefiderocol-resistant *K. pneumoniae* mutants [60,61,62]. Moreover, mutations in *fhuA* (ferrichrome iron receptor) or *fepA* (ferric enterobactin receptor) were identified in two *K. pneumoniae* clinical isolates with cefiderocol MIC of 2 mg/L [96]. 

With regard to acquired resistance to cefiderocol in *S. maltophilia*, the data available in the literature are currently very limited. However, mutations affecting iron transport (*tonB*, *exbD*, *smlat1148*, *cirA*) were reported in in vitro-derived cefiderocol-resistant *S. maltophilia* isolates [68,69].

Overall, the multitude and diversity of genes involved in iron transfer systems suggest that further studies are needed to fully understand all possible pathways leading to cefiderocol resistance and their role in combinations, including other types of resistance mechanisms.

### 3.2. Expression of β-lactamases

Overall, although cefiderocol maintains relatively good activity against most CR strains, its stability is incomplete, especially against phenotypes including NDM-producing *Enterobacterales* and *A. baumannii* and ceftazidime/avibactam-resistant KPC-producing *Enterobacterales* [23]. In addition, this finding is further supported by the enhanced activity of cefiderocol when combined with clinically available β-lactamases inhibitors and dipicolinic acid in serine-β-lactamase and MBL-producing Gram-negative bacteria, respectively [70,97]. The contribution of the various types of β-lactamases is different, as suggested by both cloning and mutagenesis experiments, as well as investigations on clinical isolates [9].

#### 3.2.1. Metallo β-lactamases

The contribution of NDM-type MBL to cefiderocol hydrolysis was shown by cloning experiments in *E. coli*, *P. aeruginosa*, and *A. baumannii* [72,73,74,75]. Four- to 64-fold increases in MIC values were observed, without significant difference among the different NDM variants (e.g., NDM-1, NDM-5, NDM-7, NDM-9) [70,71,72,73]. The combined effect of NDM expression and *cirA* deficiency in *K. pneumoniae* and *E. coli* was observed in several clinical isolates [63,64,65,66,67]. Moreover, an increased copy number of *bla*_NDM−5_ together with increased expression of *bla*_NDM−5_ was associated with reduced susceptibility to cefiderocol in *E. coli* isolates [74,75]. In addition to the NDM type, other MBLs may be implicated, although the currently available evidence is limited. In vitro studies showed that the introduction of the *bla*_SPM−1_ gene caused a significant decrease in cefiderocol activity in *E. coli* and *P. aeruginosa*, whereas VIM−2, AIM-1, and GIM-1 expression had low or no impact [72].

#### 3.2.2. KPC Variants

One of the first pieces of evidence in favor of cross-resistance between ceftazidime/avibactam and cefiderocol in KPC-producing *K. pneumoniae* (KPC-Kp) emerged by observing a significantly higher rate of cefiderocol resistance among ceftazidime/avibactam-resistant isolates than among susceptible ones (82.5% vs. 6.7%) [78]. To date, more than 200 variants of KPC have been reported worldwide, and most of them were discovered in the last five years, following the introduction of ceftazidime/avibactam intclinical practice. The sequencing of *bla*_KPC_ in clinical isolates with reduced susceptibility to cefiderocol and cloning experiments in isogenic strains confirmed that cross-resistance is due to the expression of specific KPC variants, mostly with mutations in the Ω loop of KPC (e.g., KPC-31, KPC-33, KPC-41, KPC-50, KPC-25, KPC-29, KPC-44, KPC-121, KPC-203, KPC-109, KPC-216) [76,77,79,80,81,82]. Given the structural similarity between ceftazidime and cefiderocol, mutations that increase the hydrolytic activity of the β-lactamase towards ceftazidime lead to co-resistance or reduced susceptibility to cefiderocol. Indeed, in this regard, the combination of cefiderocol with new β-lactamase inhibitors such as avibactam, vaborbactam, or relebactam seems promising to enhance its activity [97].

#### 3.2.3. OXA-type β-lactamases

The effect of OXA-type β-lactamase expression on cefiderocol resistance is limited. Cloning experiments showed that both narrow-spectrum (OXA-1) and most carbapenem-hydrolyzing (OXA-23, OXA-48, OXA-58) class D β-lactamases do not affect cefiderocol susceptibility [70,71,72,73]. In contrast, the recently identified carbapenemase OXA-427 showed significant hydrolytic activity against cefiderocol [71,72,73], although a low effect on cefiderocol MIC (two-fold increase) was demonstrated in *E. coli* isogenic mutants [72]. In addition, resistance to cefiderocol was reported in a collection of OXA-427-producing *Enterobacterales* in Belgium [83].

#### 3.2.4. AmpC β-lactamases

The involvement of ampC variants in cefiderocol resistance is demonstrated by clinical reports and cloning experiments. The A292_L293del ampC variant was associated with cross-resistance to cefepime, ceftazidime/avibactam, and cefiderocol in the *E. cloacae* complex. Cloning of the encoding gene into *E. coli* TOP10 led to resistance to cefepime and ceftazidime/avibactam and a >32-fold increase in the MIC of cefiderocol [84]. The role of four amino acid substitutions (A114E, Q120K, V211S, and N346Y) in CMY-2 ampC (CMY-185) in the development of cross-resistance to ceftazidime/avibactam and cefiderocol was also shown in an *E. coli* clinical isolate [86]. Similarly, a new variant of CMY β-lactamase (CMY-186) characterized by V159G amino acid substitution in the YXN loop of the enzyme was observed in a clinical isolate of *K. pneumoniae* [87]. 

The expression of ampC variants carrying single amino acid substitutions (E247K and L147F) was also associated with the in vivo emergence of cross-resistance to ceftolozane/tazobactam and cefiderocol in *P. aeruginosa* [53,88].

In addition, cephalosporinase PDC-30 was detected in two cases of in vivo cefiderocol-resistant *P. aeruginosa*, as the introduction of the respective gene in *E. coli* caused an eight-fold increase in the MIC of cefiderocol [17,85].

#### 3.2.5. Other β-lactamases

Several in vitro studies have demonstrated high rates of cefiderocol resistance in *A. baumannii* and *P. aeruginosa* isolates expressing PER-type β-lactamases [15,70,89]. Cloning experiments of the various *bla*_PER_-like genes in *E. coli*, *P. aeruginosa*, and *A. baumannii* isogenic mutants showed a 2- to 64-fold increase in the MIC of cefiderocol [70,71,72]. SHV-type EBSLs and BEL-type β-lactamases have been associated with increased cefiderocol MICs of cefiderocol following their introduction into isogenic mutants [70,71,72]. The amplification of *bla*_SHV-12_ has been associated with cefiderocol resistance in two hypervirulent *K. pneumoniae* clinical isolates in China [90]. 

### 3.3. Mutations in Penicillin-Binding Proteins

Since PBP-3 is the primary target of cefiderocol to exert its antibacterial effect, the presence of PBP-3 mutants represents the main mechanisms of resistance to cefiderocol at this level, although often in combination with other resistance markers (the expression of β-lactamases and/or mutations in the genes related to iron transfer systems). In detail, YRIN insertion in PBP-3 was observed in *E. coli* isolates with increased cefiderocol MIC values [62,63]. Barker et al. showed that PBP-3 mutant (*insYRIN*) alone was not sufficient to cause cefiderocol resistance but its combination with NDM expression and/or *cirA* deletion was required [66]. This evidence is supported by cloning experiments of the gene encoding the mutated PBP-3 in *E. coli*, which showed only a two-fold increase in the MIC of cefiderocol [62]. Various mutations in PBP-3 among cefiderocol-resistant *A. baumannii* clinical isolates have been identified as potential resistance mechanisms to cefiderocol [17,54,55,85]. However, their effective contribution to cefiderocol resistance needs further investigation.

### 3.4. Porin Loss or Efflux Pump Overexpression

The mechanism of porin loss has been associated with cefiderocol resistance in *K. pneumoniae* (*ompK35*, *ompK36*, *ompK37*) [91,92], *E. cloacae* (*ompC* and *ompF*) [91], and *P. aeruginosa* clinical isolates (*oprD*) [17]. Furthermore, the deletion of ompK35/ompK36 and oprD in *E. coli* and *P. aeruginosa* isogenic mutants showed a two- to four-fold increase of the MIC values of cefiderocol, respectively [50,93]. The role of efflux transport protein overexpression in cefiderocol resistance emerged in CR *K. pneumoniae* [92], *P. aeruginosa* (MexAB–OprM) [93], *S. maltophilia* (SmeDEF) [70], and *Achromobacter xylosoxidans* (AxyABM) isolates [94].

## 4. Pharmacokinetic/Pharmacodynamic (PK/PD) Features

Similar to other β-lactams, cefiderocol is a time-dependent cephalosporin characterized by linear pharmacokinetics, limited volume of distribution (approximately 15 L), short half-life (2–3 h), moderate protein binding (equal to 58%), and predominant renal clearance [98]. The fraction of dosing intervals during which the free drug concentrations exceed the MIC (%fT > MIC) represents the best PK/PD predictor of cefiderocol efficacy in infections caused by *Enterobacterales*, *P. aeruginosa*, *A. baumannii*, and *S. maltophilia* strains [99]. Notably, several preclinical models [100,101,102,103] reported that a bacteriostatic effect was observed with the attainment of a cefiderocol PK/PD target equal to 40–70%fT > MIC, whereas a bactericidal effect corresponding to ≥1-log kill reduction was reported with a cefiderocol PK/PD target of 55–88%fT > MIC. Specifically, a *P. aeruginosa* neutropenic murine thigh model found that a fT > MIC of 76.3%, 81.9%, and 88.2% was required for stasis, 1-log kill, and 2-log kill reduction in bacterial burden, respectively [102]. Similarly, in murine thigh and lung infection models, the mean %fT > MIC of cefiderocol needed for a 1-log kill reduction against ten strains of Enterobacterales and three strains of *P. aeruginosa* in the thigh infection models were 73.3% and 77.2%, respectively, whereas the mean %fT > MIC against *Enterobacteriaceae*, *P. aeruginosa*, *A. baumannii*, and *S. maltophilia* in the lung infection model were 64.4%, 70.3%, 88.1%, and 53.9%, respectively [104]. Overall, these preclinical data support the adoption of 2 g every 8 h administered over 3 h infusion for attaining the PK/PD target of 75%fT > MIC against Gram-negative pathogens showing MIC values of ≤4 mg/L [99].

However, recent preclinical and clinical studies suggested that the attainment of β-lactam aggressive PK/PD targets (i.e., at least 100% fT > 4 MIC) may be recommended for maximizing not only clinical efficacy but also for minimizing microbiological failure and suppressing resistance development [105,106]. Notably, a recent meta-analysis including 21 observational studies with a total of 4833 critically ill patients found that attaining aggressive PK/PD targets was significantly associated with a higher clinical cure rate (OR = 1.69; 95%CI 1.15–2.49) and lower risk of β-lactam resistance development (OR = 0.06; 95%CI 0.01–0.29). Conversely, failure in attaining aggressive PK/PD targets was significantly associated with a higher risk of microbiological failure (OR = 26.08; 95%CI 8.72–77.95) [106]. Although evidence concerning the adoption of aggressive PK/PD targets with novel β-lactams is currently limited, this strategy should also be pursued in this setting, including patients receiving cefiderocol.

In this regard, although evidence investigating the relationship between cefiderocol PK/PD target attainment and clinical/microbiological outcome is limited, some interesting clues have been raised. Interestingly, a recent case series of 13 critically ill patients affected by severe COVID-19 pneumonia developing CRAb bloodstream infections (BSIs) and/or ventilator-associated pneumonia treated with cefiderocol administered by extended infusion (EI) over 3 h showed that only 38% attained an aggressive PK/PD target (defined as a free trough concentration [fCmin]/MIC ratio > 4). Notably, patients attaining suboptimal PK/PD target attainment (i.e., a fCmin/MIC ratio < 1) had a higher microbiological failure rate (80%) compared with those having quasi-optimal (i.e., a fCmin/MIC ratio 1–4) or optimal PK/PD targets (29%) [107]. Similarly, the other two case series including, respectively, three patients affected by CRAb infections and receiving cefiderocol administered by 3 h EI during renal replacement therapy [108] and four cases of difficult-to-treat resistant (DTR) *P. aeruginosa* BSIs and/or nosocomial pneumonia treated with 3 h EI cefiderocol [109] reported the attainment of optimal PK/PD targets in 67% and 75% of cases, respectively.

These findings could support the rationale for implementing cefiderocol-altered dosing strategies by continuous infusion (CI) (i.e., 2 g every 8 h over 8 h infusion according to aqueous stability restriction) after proper loading (2 g over 2–3 h) for maximizing the attainment of aggressive PK/PD targets. Indeed, in a recent case series of five critically ill patients affected by severe CRAb infections and receiving CI cefiderocol (i.e., 2 g every 8 h over 8 h infusion) during continuous venovenous hemodiafiltration, aggressive PK/PD targets were attained in all included patients, ensuring optimal coverage for MIC up to 4 mg/L included [110].

It is noteworthy that optimal penetration and consequent attainment of aggressive PK/PD targets in infection sites represent a mandatory goal in the treatment of deep-seated infections, as suggested by the “antimicrobial puzzle” concepts [111]. In this scenario, evidence concerning cefiderocol penetration and absolute tissue concentrations are available for the lungs, central nervous system (CNS), and abdominal fluid.

Specifically, a prospective observational study investigating epithelial lining fluid (ELF) penetration of cefiderocol administered at a dosage of 2 g every 8 h by EI in 7 critically ill patients affected by ventilator-associated pneumonia reported a mean ELF-to-plasma ratio of 0.34, being the attainment of aggressive PK/PD targets ensured only for MIC up to 0.5 mg/L [112,113].

Four case reports assessed penetration rate and absolute concentrations of cefiderocol in cerebrospinal fluid (CSF) [114]. Specifically, the cefiderocol CSF-to-plasma ratio ranged from 0.04 to 0.50, whereas absolute fCmin ranged from 0.65 mg/L to 10.46 mg/L with administration of 2 g every 6–8 h over 3 h EI. Although retrieved concentrations allowed us to attain the optimal cefiderocol PK/PD target in three out of four cases according to the MIC values of isolated pathogens, only in one case did concentrations ensure the attainment of the optimal PK/PD target for MIC up to the clinical breakpoint of 2 mg/L [114].

In regard to abdominal fluid penetration, a case report of a critically ill patient affected by DTR *P. aeruginosa* infection and treated with CI cefiderocol during continuous venovenous hemofiltration reported absolute concentrations in abdominal drain fluid equal to 15.3 mg/L, ensuring the attainment of optimal PK/PD target for MIC values up to the clinical breakpoint of 2 mg/L [115].

Overall, these findings may strongly support the adoption of cefiderocol-altered dosing strategies consisting of a high dosage coupled with CI administration for maximizing the attainment of optimal PK/PD targets in case of deep-seated infections [116].

## 5. Clinical Usage

Infections caused by GNB-MDR and, in particular, by extensively drug-resistant-(XDR)GNB, present a challenge to clinicians due to the high mortality rate of up to 30–50%, especially in critically ill patients, because of limited antimicrobial options against these microorganisms. [3,4]. In this context, the availability of cefiderocol represented a crucial innovation for clinicians. As discussed above, cefiderocol was approved in 2019 by the FDA for the treatment of complicated urinary tract infections (cUTIs) [117] and for hospital-acquired pneumonia (HAP) and ventilator-acquired pneumonia (VAP) based on the CREDIBLE-CR, APEKS cUTI, and APEKS NP phase III clinical trials [18,118]. Then, the EMA approved cefiderocol for the treatment of infections caused by GNB with limited therapeutic options, including CRPa, *S. maltophilia*, CRE, and CRAb [119].

Overall, considering the initial approval prescription limitations, cefiderocol has been subsequently approved for the treatment of severe infections due to CR-GNB (Table 3). 

### 5.1. Carbapenem-Resistant Pseudomonas aeruginosa 

*P. aeruginosa* is provided with different mechanisms of antibiotic resistance, and the prevalence of CRPa strains ranged between 10 and 25% in most European countries [135]. At the same time, several studies reported that the emergence of novel traits of resistance to newer BL-BLICs in CRPa is spreading, while cefiderocol seems to retain sensitivity against CRPa strains [136]. In a recent study, de La Fuente et al. reported a 28-day crude mortality of 23%, with an achievement of clinical and microbiological cure of 84.6% (assessed 7 days after the end of treatment) [120] in a cohort of 13 patients mainly affected by CRPa pneumonia. A larger real-world case series published by Gavaghan et al. reported a clinical success of 46% and a30-day mortality of 42% in 24 patients with CR-GNB infections (mainly CRAb at 14.5% and CRPa at 10.4%) treated with cefiderocol [121].

A recent study conducted among 27 ICU patients affected by non-fermenting GNB infections (*P. aeruginosa* in 81.5% of cases) treated with cefiderocol compared to 54 patients who received the best available therapy (BAT) showed a higher relapse rate in cefiderocol with a comparable result in clinical cure or mortality rates compared to BAT [122].

A recent retrospective multi-center study showed an overall clinical success rate of 84.3% at day 28 and 28-day all-cause mortality of 21.5% in patients with GNB infection, mainly *P. aeruginosa* (66.7%), treated with cefiderocol [127].

### 5.2. Stenotrophomonas maltophilia

Based on the literature review, the mortality rates associated with severe infections due to *S. maltophilia* ranged between 21 and 69% [137,138], and studies regarding the efficacy of cefiderocol against patients with infections due to *S. maltophilia* are scarce. However, a recent study conducted by Cai et al. showed 36.8% and 21% in-hospital all-cause mortality at 14 days and 28 days, respectively [123].

### 5.3. Carbapenem-Resistant Enterobacterales (CRE)

A subanalysis of the results of a CREDIBLE-CR and APEKS-NP trial including infections due to MBL-producers gave us encouraging results, with higher rates of clinical cure, microbiological eradication, and 28-day all-cause mortality compared to BAT or high-dose meropenem, respectively [124]. In a real-world case series including 21 patients with infections caused by MBL-producing CRE, the rate of cefiderocol resistance was 9.5% [125]. Indeed, according to microbiological data, cefiderocol resistance rates among NDM strains are 81% and 12% using EUCAST and CLSI interpretive criteria, respectively [139].

According to these data, the empirical use of cefiderocol for the treatment of MBL-*Enterobacterales* infections should be discouraged until the results of a microbiological susceptibility test.

### 5.4. Carbapenem-Resistant Acinetobacter baumannii (CRAb)

Several case series and comparative studies explored the efficacy and safety of this new drug in the “real-world”. In particular, Bavaro et al. reported a case series of 13 patients treated with cefiderocol-based combination therapy (10/13) against CRAb with a rate of microbiological eradication of 100% and a 30-day survival rate of 77% [126]. Similarly, another interesting case series including 10 critically ill patients with bloodstream infection (BSI) or VAP mainly caused by CRAb stated a clinical success of 70%, while 30-day mortality was 10% [127]. Another study including 118 patients with CRAb monomicrobial bacteremia showed significantly lower 30-day mortality in patients treated with cefiderocol compared to patients treated with colistin (40% vs. 59%, *p* = 0.045), and cefiderocol-based treatment had protective results [128].

Dalfino et al. explored the difference in terms of clinical failure between patients affected by CRAb VAP treated with cefiderocol-based regimens and colistin-based regimens: a higher Charlson comorbidity index independently predicted the occurrence of clinical failure, while timely targeted antibiotic treatment and cefiderocol-based first-line regimens reduced the risk of treatment failure [129]. Further studies highlighted how the use of cefiderocol for treatment of VAP caused by CRAb was associated with a benefit in 28-day [130] and 30-day mortality [131]. An additional study including patients affected by CRAb BSI treated with cefiderocol or colistin showed a significantly higher clinical cure in the cefiderocol group and a lower rate of adverse events; particularly, in HAP/VAP group treatment, cefiderocol was associated with lower 30-day mortality and more clinical cures [132].

In an Italian multicentric retrospective study by Pascale et al., 107 patients admitted to the ICU for COVID-19 who developed CRAb infection treated with cefiderocol as monotherapy in 45% of cases and colistin-based regimens in 55%, showed no significant difference in the all-cause 28-day mortality rate between the two groups; however, in a multivariable analysis, cefiderocol was associated with a non-significant lower mortality risk [133].

The ARES study, a multicentric retrospective cohort study including critically ill patients treated with cefiderocol for CRAb infection, resulted in a clinical success rate of 53%, with better results in subjects who were not affected by septic shock and COVID-19, and with a lower SOFA score [134].

Overall, the results of real-world studies are more encouraging if compared to CREDIBLE-CR, where cefiderocol was non-inferior when compared to the best available therapy in terms of clinical and microbiological response but resulted in a higher mortality rate (49% vs. 18%), with the majority of deaths in the CRAb group. Notably, the cefiderocol group included patients with higher SOFA and CCI scores who experienced previous failure to BAT.

On the basis of the current available data, the latest Infectious Diseases Society of America (IDSA) guidance for the treatment of antimicrobial-resistant Gram-negative infections recommends limiting the use of cefiderocol as salvage therapy for CRAb infections, only in cases of resistance or intolerance to other regimens, as part of a combination therapy [140].

## 6. Prospectives and Open Questions

Based on the literature review, several questions regarding the clinical use of cefiderocol and, in particular, its use as monotherapy or as part of combination regimes remain open and unresolved.

Regarding combination therapy, despite its usefulness in clinical trials not being demonstrated, this strategy could be successful in clinical practice for different reasons: (i) the synergistic effect, (ii) to avoid resistance development, and (iii) to extend the spectrum of antimicrobial activity in the first phase of treatment when the causative pathogen and its sensitivity profile are still unknown. In this sense, a good partner for cefiderocol could be intravenous fosfomycin, even for CRAb, which is intrinsically resistant to the epoxide molecule [141]; indeed, its mechanism of action leads to a stressful condition that makes pathogens more sensitive to other antimicrobial agents and prevents the development of resistance [142].

Despite resistance to cefiderocol not being frequently detected, it should be taken into account when a susceptibility test is not available. As discussed above, multiple mechanisms contribute to this condition: increased expression of beta-lactamase (metallo beta lactamase), mutation of the target protein or siderophore receptor and outer membrane porins, and overexpression of efflux transport proteins [9,143].

Moreover, the increase in resistance in the course of treatment has been described in several studies. In particular, among other reasons, the phenomenon of hetero-resistance could be involved in this process; it occurs when a small percentage of bacteria develop resistance under the pressure of antibiotics. Consequently, the non-susceptible population grows during prolonged exposure to antibiotics [83,89,144,145,146].

Combination therapy could play a role in reducing this phenomenon, along with the optimization of the pharmacokinetic/pharmacodynamic parameters of cefiderocol [147].

Despite the innovativeness of this molecule, the encouraging results of a real-world study in terms of safety and effectiveness, and the need for new antibiotics for the treatment of XDR-GNB, some open questions about its use in daily clinical practice remain. The prescription of cefiderocol in hospital settings where the rate of CR-GNB is high could be considered in case of severe infections and septic shock, in the absence of other therapeutic chances, in order to promptly provide effective therapy. Further clinical trials to optimize the use of cefiderocol are warranted.

## 7. Conclusions

The limited antimicrobial options for the treatment of infections due to MDR microorganisms pose an urgent need for new antimicrobial drugs. To combat this scourge and counter the increasing spread of antimicrobial resistance in Gram-negative bacteria, the development of new antimicrobial molecules has been proposed in recent years. In this context, cefiderocol represents a new valuable option for the treatment of infections due to MDR pathogens. Its high antibacterial activity, excellent safety profile, tolerability, and broad spectrum of activity against various Gram-negative pathogens have earned cefiderocol the role of “*promising antibiotic*” for the treatment of MDR bacteria, laying the foundations for new antimicrobial weapons and defining new paradigms for the treatment of infections, especially in critically ill patients.

However, despite the initial promising in vitro and in vivo results, the emergence of resistance to cefiderocol has been observed worldwide, with an increasing number of clinical reports only four years after its introduction into clinical use. Based on these conflicting aspects, further investigations are needed to identify the role and diversity of the siderophore receptor repertoire in Gram-negative bacteria in order to better understand the mechanisms of resistance induction. At the same time, several points need to be addressed to better define the role of this novel molecule and to preserve its activity against different MDR Gram-negative bacteria.

## Figures and Tables

**Table 1:** In vitro activity of cefiderocol against major Gram-negative bacilli collections according to EUCAST or CLSI breakpoints.

Ref.	Country	Study Remarks	Guidelines	Overall Isolates							Carbapenem-Non-Susceptible						Carbapenemase Producers		
				EB	PA	ACB	STEMA	ACHR	BURK		EB	PA	ACB	ACHR	BURK		EB	PA	ACB
[3]	Worldwide	Meta-analysis	EUCAST	97	98.6	91.2	99.6				87.6	96.5	86.8				61.2–75.1	77.1–98.2	55.3–59.1
[4]	India	K. pneumoniae	CLSI	82.3							80.6								
[5]	Spain, Portugal		CLSI	99.5	100	98.4	100												
[6]	UAE	K. pneumoniae	CLSI	97.9							93.3								
[7]	China	K. pneumoniae	CLSI	95.6							90.3						86–90		
[8]	Czech Republic		EUCAST								82	84							
[9]	India		CLSI								93.3	95.2	100						
[10]	India		CLSI								97.2	100	93.4						
[11]	USA		CLSI		93.8														
[12]	USA		CLSI		95.6							93.3							
[13]	USA	CF patients	CLSI		80.7							63							
[14]	Worldwide		EUCAST		95													69–100	
[15]	Europe		EUCAST		98.9	92.4						97.8	85						
[16]	Spain	CF patients	EUCAST		91.5							70.6							
[17]	Italy		EUCAST		95.8														
[18]	Taiwan		CLSI			94.4	97.9					100			100				
[19]	UK	CF patients	EUCAST				86.2	87.8	70.7										
[20]	Italy		EUCAST				100						96	97.2			83.4–87.5		
[21]	Mexico		CLSI				95.1												
[22]	Worldwide		EUCAST					96.7	94.8					88.5	92.9				
[23]	France, Denmark	CF patients	EUCAST					99.1						96.3					
[24]	India		CLSI						100										
[25]	Switzerland	MBL producers	EUCAST															79.5	
[26]	Turkey												59.6						
[27]	USA		CLSI										96.9						97.9
[28]	Japan		CLSI														83.3–100	100	91.2
[29]	Spain	*E. coli*	EUCAST														92.2		

Grey shading indicates cefiderocol activity <90%. Abbreviations: EB: Enterobacterales; PA: *Pseudomonas aeruginosa*; ACB: *Acinetobacter baumannii complex*; STEMA: *Stenotrophomonas maltophilia*; ACHR: *Achromobacter* species; BURK: *Burkholderia* species; MBL: metallo-β-lactamase; CF, Cystic fibrosis.

**Table 2:** Mechanisms associated with reduced susceptibility or resistance to cefiderocol.

Mechanism	Bacterial Species		Genes	Combination of Resistance Mechanisms
Mutations in genes related to iron transfer systems	*P. aeruginosa* *A. baumanii*	In vitro-induced mutants or isogenic strains	*piuA* [50,51], *piuD* [51], *pirA* [50,51]	*piuA* or *piuD* with deletion of *pirA*—the increase was 32-fold and 64-fold, respectively [50,51].
		Clinical isolates	*piuDC* [52], *piuA* [53,54,55,56], *pirR* [53], *pirA* [54,57]	Deletion of *piuDC* and the ampC regulator ampD led to co-resistance to cefiderocol and ceftazidime/avibactam in *P. aeruginosa* [52]. Substitution of L147F in ampC gene together with mutations in *piuA* and *pirR* genes led to acquired resistance to cefiderocol and ceftolozane/tazobactam in *P. aeruginosa* [53]. *piuA* deficiency together with reduced expression of *pirA* in cefiderocol-resistant *A. baumannii* clinical isolate [54].
	Enterobacterales	In vitro-induced mutants or isogenic strains	*cirA* [58,59], *fiu* [50], *tonB*, *exbB*, *exbD*, *fecA*, *fbpA*, *efeo* [60,61,62]	Loss of *fiu* resulted in two-fold increase of cefiderocol MIC, increasing up to 16-fold when combined with deletion of *cirA* [50].
		Clinical isolates	*CirA* [63,64,65,66,67]	Most cefiderocol-resistant *K. pneumoniae* or *E. coli* clinical isolates presenting mutations in *cirA* were also producers of NDM metallo β-lactamase [63,64,65,66,67].
	*S. maltophilia*	In vitro-induced mutants or isogenic strains	*tonB*, *exbD*, *smlat1148*, *cirA* [68,69]	
Expression of metallo-β-lactamases	Enterobacterales *P. aeruginosa*	In vitro-induced mutants or isogenic strains	*bla_NDM-type_* [70,71,72,73], *bla*_SPM−1_ [72]	≥8-fold increase of cefiderocol MIC was observed when both serine- and metallo-type β-lactamase inhibitors were added in *E. coli* isogenic strains [70].
	Enterobacterales	Clinical isolates	*bla_NDM-type_* [63,64,65,66,67,70,74,75]	The combined effect of NDM expression and *cirA* deficiency in *K. pneumoniae* and *E. coli* was associated with cefiderocol resistance [63,64,65,66,67,74].
Expression of KPC variants	Enterobacterales	In vitro-induced mutants or isogenic strains	*bla_KPC−31_*, *bla_KPC−33_*, *bla_KPC−25_*, *bla_KPC−29_*, *bla_KPC−39_*, *bla_KPC−44_*, *bla_KPC−41_*, *bla_KPC−50_* [76,77]	
	*K. pneumoniae*	Clinical isolates	*bla_KPC−31_*, *bla_KPC−33_*, *bla_KPC−41_*, *bla_KPC−50_*, *bla_KPC−121_*, *bla_KPC−109_*, *bla_KPC−203_*, *bla_KPC−216_* [77,78,79,80,81,82]	
Expression of OXA-like β-lactamases	*A. baumannii*, *E. coli*, *P. aeruginosa*	In vitro-induced mutants or isogenic strains	*bla*_OXA−427_ [71,72,83]	
	Enterobacterales	Clinical isolates	*bla*_OXA−427_ [83]	
Expression of ampC variants	*E. coli*	In vitro-induced mutants or isogenic strains	A292_L293del *bla_ampC_* [84] *bla*_PDC−30_ [85]	
	*E. cloacae* complex, *E. coli*, *P. aeruginosa*	Clinical isolates	A292_L293del *bla_ampC_* [84], *bla*_CMY−185_ [86] *bla*_CMY−186_ [87], L147F *bla_PDC−191_* [53] E247K *bla_PDC-like_* [88] *bla*_PDC−30_ [85]	SNPs identified in genes belonging to TonB-dependent receptors (TBDRs) and in the chromosomal *ampC* β-lactamase gene were associated with in vivo emergence of cefiderocol resistance in *P. aeruginosa* [53]. Mutations in ampD G116D and in efflux pump system (MexR A66V, MexR L57D) were associated with increases in cefiderocol MICs in *P. aeruginosa* [88].
Expression of other β-lactamases	*E. coli*, *P. aeruginosa*, *A. baumannii*,	In vitro-induced mutants or isogenic strains	*bla_PER-like_* [70,71,72]	
	*K. pneumoniae*	Clinical isolates	*bla_PER−1_* [70], *bla_PER-like_* [89], amplification of *bla*_SHV−12_ [90]	
Mutations in penicillin-binding proteins	*A. baumanni*, *E. coli*	Clinical isolates	PBP−3 gene (*ftsI)* [17,54,55,62,63,85]	PBP-3 mutant (insYRIN) alone was not sufficient to cause cefiderocol resistance in *E. coli*, but its combination with NDM expression and/or *cirA* deletion was required [66]. Combination of NDM-5 expression with mutated *ftsI and cirA genes was observed in cefiderocol resistance E. coli isolates* [63].
Porin loss	*K. pneumoniae*, *E. cloacae*, *P. aeruginosa*	Clinical isolates	*ompK35*, *ompK36*, *ompK37* [91,92], *ompC* and *ompF* [91], *oprD* [17]	
Efflux pump overexpression	*P. aeruginosa*, *S. maltophilia*, *K. pneumoniae*, *A. xylosoxidans*	Clinical isolates	*mexAB–oprM* [93], *smeDEF* [70], *sugE*, *chrA* [92] *axyABM* [94]	

**Table 3:** Clinical use, clinical outcomes, and microbiological outcomes of cefiderocol.

Microbiological Outcomes	Clinical Outcomes	Clinical Characteristics	Studies	
Microbiological cure: 87%	Clinical cure: 87%, 28-day mortality: 25%	8 CR Pa infections -> 5 pneumonia, 1 pneumonia + BSI, 2 IAIs	de La Fuente et al. [120]	CR-P. aeruginosa
Infection recurrence: 40%	Clinical cure: 60%, death: 30%	10 CR-Pa infections (6 monomicrobial, 4 polymicrobial) -> 7 pneumonia, 2 BSIs, 1 wound	Gavagan et al. [121]	
Relapse: 30%	Clinical cure at 30 days: 44%, in ICU mortality: 52%	22 CR-Pa infections in ICU, mostly pneumonia	Vacheron et al. [122]	
N/A	Crude overall in-hospital all-cause mortality: 36.8%	19 patients -> 68% pneumonia, 10% BSIs, 5% urinary, 5% wounds	Cai et al. [123]	S. maltophilia
Microbiological cure: CFD = 58.3%,BAT = 30%	Clinical cure: CFD = 70.8%, BAT = 40%; 28-day all-cause mortality: CFD = 12.5%, BAT = 50%	24 patients affected by MBL-producing GNB infections from CREDIBLE-CR and APEKS-NP studies	Timsit et al. [124]	CR-Enterobacterales
Eradication at end of treatment: 77.8%	Clinical cure: 72.2%, 28-day all-cause mortality: 22.2%	18 patients affected by MBL-producing GNB infections	Falcone et al. [125]	
Infection recurrence: 21%	Clinical cure: 36%, death: 43%	14 CRAb infections (5 polymicrobial) -> 10 pneumonia, 2 pneumonia + BSI, 1 UTI, 1 wound + BSI	Gavagan et al. [121]	CR-A. baumannii
Microbiological cure: 100%	30-day survival rate: 77%	10 monomicrobial CRAb BSIs	Bavaro et al. [126]	
Recurrence: 25%	Clinical success: 75%, 30-day mortality: 12%	8 CRAb infections -> 6 BSI, 2 VAP	Falcone et al. [127]	
90-day infection recurrence/relapse: CFD = 9% vs. colistin = 5%	30-day infection-related mortality: CFD = 30% vs. colistin 56%	118 monomicrobial CRAb BSIs, 75 treated with colistin vs. 43 treated with CFD	Bavaro et al. [128]	
Microbiological failure: CFD= 30% vs. colistin = 60%	Clinical failure: CFD = 25% vs. colistin = 48%; 14-day mortality: CFD = 10% vs. colistin = 38%; 28-day mortality: CFD = 35% vs. colistin = 52%	90 monomicrobial VAP, 40 treated with cefiderocol vs. 50 treated with colistin (bacteraemic 33% vs. 32%)	Dalfino et al. [129]	
Microbiological failure: CFD = 53%, non-CFD = 31%	28-day mortality: CFD = 44% vs. non-CFD = 67%	121 VAP, CFD vs. non-CFD = 55 vs. 66 (17% bacteraemic)	Rando et al. [130]	
N/A	14-day mortality: CFD= 5% vs. non-CF= 75%; 30-day mortality: CFD = 31% vs. non-CFD = 98%	73 bacteraemic VAP, 19 treated with CFD-basedregimens	Russo et al. [131]	
Microbiological eradication: CFD = 72% vs. colistin = 66%; relapse: CFD = 8% vs. colistin = 9%	Clinical cure: CFD= 66% vs. colistin = 44%; 14-day mortality: CFD = 22% vs. colistin = 31%; 30-day mortality: CFD = 36% vs. colistin = 43%	104 CRAb-BSI, 50 CFD vs. 54 colistin	Oliva et al. [132]	
14-day microbiological cure: CFD = 28%, non-CFD = 21%	14-day clinical cure: CFD = 40% vs. non-CFD = 36%; 14-day mortality: CFD = 40% vs. non-CFD = 51%	107 CRAb infections in patients with severe COVID-19, 42 treated with CFD	Pascale et al. [133]	
Relapse of primary Acinetobacter spp. infection: 10.2%	Clinical success rate: 53.1%	147 CRAb infections -> pneumonia: 65.3%, primary BSI: 15.6%, catheter-related: 10.9%, polymicrobial: 24.5%	Giannella et al. [134]	

**Abbreviations: CR-P. aeruginosa:** carbapenem-resistant Pseudomonas aeruginosa. **CR-Enterobacterales**: carbapenem-resistant Enterobacterales. **CR-A. baumannii:** carbapenem-resistant Acinetobacter baumannii. **BSIs:** bloodstream infections. **IAIs:** intrabdominal infections. **N/A:** not available. **UTI:** urinary tract infections. **VAP =** ventilator-associated pneumonia. **CFD =** cefiderocol.

## Data Availability

Not applicable.

## References

[1] Bloom D.E., Black S., Salisbury D., Rappuoli R. (2018). Antimicrobial resistance and the role of vaccines. Proc. Natl. Acad. Sci. USA.

[2] Shi Z., Zhang J., Tian L., Xin L., Liang C., Ren X., Li M. (2023). A Comprehensive Overview of the Antibiotics Approved in the Last Two Decades: Retrospects and Prospects. Molecules.

[3] Bassetti M., Kanj S.S., Kiratisin P., Rodrigues C., Van Duin D., Villegas M.V., Yu Y. (2022). Early appropriate diagnostics and treatment of MDR Gram-negative infections. JAC-Antimicrob. Resist..

[4] Parmanik A., Das S., Kar B., Bose A., Dwivedi G.R., Pandey M.M. (2022). Current Treatment Strategies Against Multidrug-Resistant Bacteria: A Review. Curr. Microbiol..

[5] Boyd S.E., Livermore D.M., Hooper D.C., Hope W.W. (2020). Metallo-β-Lactamases: Structure, Function, Epidemiology, Treatment Options, and the Development Pipeline. Antimicrob. Agents Chemother..

[6] Echols R., Ariyasu M., Nagata T.D. (2019). Pathogen-focused Clinical Development to Address Unmet Medical Need: Cefiderocol Targeting Carbapenem Resistance. Clin. Infect. Dis..

[7] Heil E.L., Tamma P.D. (2021). Cefiderocol: The Trojan horse has arrived but will Troy fall?. Lancet Infect. Dis..

[8] Abdul-Mutakabbir J.C., Alosaimy S., Morrisette T., Kebriaei R., Rybak M.J. (2020). Cefiderocol: A Novel Siderophore Cephalosporin against Multidrug-Resistant Gram-Negative Pathogens. Pharmacotherapy.

[9] Karakonstantis S., Rousaki M., Kritsotakis E.I. (2022). Cefiderocol: Systematic Review of Mechanisms of Resistance, Heteroresistance and In Vivo Emergence of Resistance. Antibiotics.

[10] Ong’uti S., Czech M., Robilotti E., Holubar M. (2022). Cefiderocol: A New Cephalosporin Stratagem Against Multidrug-Resistant Gram-Negative Bacteria. Clin. Infect. Dis..

[11] Karlowsky J.A., Hackel M.A., Takemura M., Yamano Y., Echols R., Sahm D.F. (2022). In Vitro Susceptibility of Gram-Negative Pathogens to Cefiderocol in Five Consecutive Annual Multinational SIDERO-WT Surveillance Studies, 2014 to 2019. Antimicrob. Agents Chemother..

[12] Candel F.J., Henriksen A.S., Longshaw C., Yamano Y., Oliver A. (2022). In vitro activity of the novel siderophore cephalosporin, cefiderocol, in Gram-negative pathogens in Europe by site of infection. Clin. Microbiol. Infect..

[13] Golden A.R., Adam H.J., Baxter M., Walkty A., Lagacé-Wiens P., Karlowsky J.A., Zhanel G.G. (2020). In Vitro Activity of Cefiderocol, a Novel Siderophore Cephalosporin, against Gram-Negative Bacilli Isolated from Patients in Canadian Intensive Care Units. Diagn. Microbiol. Infect. Dis..

[14] Morris C.P., Bergman Y., Tekle T., Fissel J.A., Tamma P.D., Simner P.J. (2020). Cefiderocol Antimicrobial Susceptibility Testing against Multidrug-Resistant Gram-Negative Bacilli: A Comparison of Disk Diffusion to Broth Microdilution. J. Clin. Microbiol..

[15] Mushtaq S., Sadouki Z., Vickers A., Livermore D.M., Woodford N. (2020). In Vitro Activity of Cefiderocol, a Siderophore Cephalosporin, against Multidrug-Resistant Gram-Negative Bacteria. Antimicrob. Agents Chemother..

[16] Choby J.E., Ozturk T., Satola S.W., Jacob J.T., Weiss D.S. (2021). Widespread cefiderocol heteroresistance in carbapenem-resistant Gram-negative pathogens. Lancet Infect. Dis..

[17] Takemura M., Yamano Y., Matsunaga Y., Ariyasu M., Echols R., Nagata T.D. (2020). 1266. Characterization of Shifts in Minimum Inhibitory Concentrations During Treatment with Cefiderocol or Comparators in the Phase 3 CREDIBLE-CR and APEKS-NP Studies. Open Forum Infect. Dis..

[18] Wunderink R.G., Matsunaga Y., Ariyasu M., Clevenbergh P., Echols R., Kaye K.S., Kollef M., Menon A., Pogue J.M., Shorr A.F. (2020). Cefiderocol versus high-dose, extended-infusion meropenem for the treatment of Gram-negative nosocomial pneumonia (APEKS-NP): A randomised, double-blind, phase 3, non-inferiority trial. Lancet Infect. Dis..

[19] Bassetti M., Echols R., Matsunaga Y., Ariyasu M., Doi Y., Ferrer R., Lodise T.P., Naas T., Niki Y., Paterson D.L. (2021). Efficacy and safety of cefiderocol or best available therapy for the treatment of serious infections caused by carbapenem-resistant Gram-negative bacteria (CREDIBLE-CR): A randomised, open-label, multicentre, pathogen-focused, descriptive, phase 3 trial. Lancet Infect. Dis..

[20] Klein S., Boutin S., Kocer K., Fiedler M.O., Störzinger D., Weigand M.A., Tan B., Richter D., Rupp C., Mieth M. (2021). Rapid Development of Cefiderocol Resistance in Carbapenem-resistant Enterobacter cloacae During Therapy Is Associated with Heterogeneous Mutations in the Catecholate Siderophore Receptor cirA. Clin. Infect. Dis..

[21] Aoki T., Yoshizawa H., Yamawaki K., Yokoo K., Sato J., Hisakawa S., Hasegawa Y., Kusano H., Sano M., Sugimoto H. (2018). Cefiderocol (S-649266), A new siderophore cephalosporin exhibiting potent activities against Pseudomonas aeruginosa and other gram-negative pathogens including multi-drug resistant bacteria: Structure activity relationship. Eur. J. Med. Chem..

[22] Sato T., Yamawaki K. (2019). Cefiderocol: Discovery, Chemistry, and In Vivo Profiles of a Novel Siderophore Cephalosporin. Clin. Infect. Dis..

[23] Karakonstantis S., Rousaki M., Vassilopoulou L., Kritsotakis E.I. (2024). Global prevalence of cefiderocol non-susceptibility in Enterobacterales, *Pseudomonas aeruginosa*, *Acinetobacter baumannii*, and *Stenotrophomonas maltophilia*: A systematic review and meta-analysis. Clin. Microbiol. Infect..

[24] Borde K., Borde K., Kareem M.A., Kareem M.A., Sharma R.M., Sharma R.M., Dass S.M., Dass S.M., Ravi V., Ravi V. (2023). In vitro activity of cefiderocol against comparators (ceftazidime-avibactam, ceftazidime-avibactam/aztreonam combination, and colistin) against clinical isolates of meropenem-resistant *Klebsiella pneumoniae* from India. Microbiol. Spectr..

[25] Gijón D., García-Castillo M., Fernández-López M.d.C., Bou G., Siller M., Calvo-Montes J., Pitart C., Vila J., Torno N., Gimeno C. (2024). In vitro activity of cefiderocol and other newly approved antimicrobials against multi-drug resistant Gram-negative pathogens recovered in intensive care units in Spain and Portugal. Rev. Esp. Quimioter..

[26] Daoud L., Al-Marzooq F., Ghazawi A., Anes F., Collyns T. (2023). High efficacy and enhanced synergistic activity of the novel siderophore-cephalosporin cefiderocol against multidrug-resistant and extensively drug-resistant Klebsiella pneumoniae from inpatients attending a single hospital in the United Arab Emirates. J. Infect. Public Health.

[27] Zhao J., Pu D., Li Z., Liu X., Zhang Y., Wu Y., Zhang F., Li C., Zhuo X., Lu B. (2023). In vitro activity of cefiderocol, a siderophore cephalosporin, against carbapenem-resistant hypervirulent *Klebsiella pneumoniae* in China. Antimicrob. Agents Chemother..

[28] Malisova L., Vrbova I., Pomorska K., Jakubu V., Zemlickova H. (2023). In Vitro Activity of Cefiderocol Against Carbapenem-Resistant *Enterobacterales* and *Pseudomonas aeruginosa*. Microb. Drug Resist..

[29] Khanchandani H., Chaudhury M., Rao M.S., Ramakrishna N., Venkataramana B., Chaudhury A. (2024). In vitro activity of the newly approved antimicrobial agent Cefiderocol against Carbapenem resistant Gram negative clinical isolates. Indian J. Med. Microbiol..

[30] Saxena S., Aggarwal P., Mitra S., Singh S., Kaim M., Sharma A. (2024). In vitro assessment of newer colistin-sparing antimicrobial agents for clinical isolates of carbapenem-resistant organisms. J. Infect. Chemother..

[31] Shields R.K., Kline E.G., Squires K.M., Van Tyne D., Doi Y. (2023). In vitro activity of cefiderocol against *Pseudomonas aeruginosa* demonstrating evolved resistance to novel β-lactam/β-lactamase inhibitors. JAC-Antimicrob. Resist..

[32] Riccobene T., Ai C., Yu K.C., Gregory S., Kim B., Debabov D., Gupta V. (2023). Real-world *in vitro* activity of newer antibiotics against Enterobacterales and *Pseudomonas aeruginosa*, including carbapenem-non-susceptible and multidrug-resistant isolates: A multicenter analysis. Microbiol. Spectr..

[33] Monogue M.L., Desai D., Pybus C.A., Sanders J.M., Clark A.E., Greenberg D.E. (2023). In vitro activity of cefiderocol against *Pseudomonas aeruginosa* isolated from cystic fibrosis patients. Microbiol. Spectr..

[34] Gill C.M., Santini D., Nicolau D.P. (2024). In vitro activity of cefiderocol against a global collection of carbapenem-resistant *Pseudomonas aeruginosa* with a high level of carbapenemase diversity. J. Antimicrob. Chemother..

[35] Henriksen A.S., Jeannot K., Oliver A., Perry J.D., Pletz M.W., Stefani S., Morrissey I., Longshaw C. (2024). In vitro activity of cefiderocol against European *Pseudomonas aeruginosa* and *Acinetobacter* spp., including isolates resistant to meropenem and recent β-lactam/β-lactamase inhibitor combinations. Microbiol. Spectr..

[36] Maruri-Aransolo A., López-Causapé C., Hernández-García M., García-Castillo M., Caballero-Pérez J.d.D., Oliver A., Cantón R. (2024). In vitro activity of cefiderocol in *Pseudomonas aeruginosa* isolates from people with cystic fibrosis recovered during three multicentre studies in Spain. J. Antimicrob. Chemother..

[37] Valzano F., La Bella G., Lopizzo T., Curci A., Lupo L., Morelli E., Mosca A., Marangi M., Melfitano R., Rollo T. (2024). Resistance to ceftazidime–avibactam and other new β-lactams in *Pseudomonas aeruginosa* clinical isolates: A multi-center surveillance study. Microbiol. Spectr..

[38] Huang Y.-S., Chuang Y.-C., Chen P.-Y., Chou P.-C., Wang J.-T. (2024). In vitro activity of cefiderocol and comparator antibiotics against multidrug-resistant non-fermenting Gram-negative bacilli. JAC-Antimicrob. Resist..

[39] Tunney M.M., Elborn J.S., McLaughlin C.S., Longshaw C.M. (2024). In vitro activity of cefiderocol against Gram-negative pathogens isolated from people with cystic fibrosis and bronchiectasis. J. Glob. Antimicrob. Resist..

[40] Bianco G., Boattini M., Comini S., Iannaccone M., Casale R., Allizond V., Barbui A.M., Banche G., Cavallo R., Costa C. (2022). Activity of ceftolozane-tazobactam, ceftazidime-avibactam, meropenem-vaborbactam, cefiderocol and comparators against Gram-negative organisms causing bloodstream infections in Northern Italy (2019–2021): Emergence of complex resistance phenotypes. J. Chemother..

[41] Méndez-Sotelo B.J., Delgado-Beltrán M., Hernández-Durán M., Colín-Castro C.A., Esquivel-Bautista J., Ortega-Oliva S.A., Ortiz-Álvarez J., García-Contreras R., Franco-Cendejas R., Jacome L.E.L. (2024). In vitro activity of ceftazidime/avibactam, cefiderocol, meropenem/vaborbactam and imipenem/relebactam against clinical strains of the Stenotrophomonas maltophilia complex. PLoS ONE.

[42] Takemura M., Nakamura R., Ota M., Nakai R., Sahm D.F., Hackel M.A., Yamano Y. (2023). In vitro and in vivo activity of cefiderocol against *Achromobacter* spp. and *Burkholderia cepacia* complex, including carbapenem-non-susceptible isolates. Antimicrob. Agents Chemother..

[43] Jean-Pierre V., Sorlin P., Pantel A., Chiron R., Lavigne J.-P., Jeannot K., Marchandin H., Amara M., Cadot L., Collaborative study group on antimicrobial resistance of Achromobacter spp. (2024). Cefiderocol susceptibility of *Achromobacter* spp.: Study of an accurately identified collection of 230 strains. Ann. Clin. Microbiol. Antimicrob..

[44] Jena J., Behera B., Nayak G., Mohanty S., Mahapatra A., Purushotham P., Radhakrishnan A., Tripathy M. (2023). In Vitro Susceptibility of *Burkholderia pseudomallei* Isolates to Cefiderocol and Ceftazidime/Avibactam from Odisha, India. J. Lab. Physicians.

[45] Findlay J., Raro O.H.F., Poirel L., Nordmann P. (2024). Molecular analysis of metallo-beta-lactamase-producing *Pseudomonas aeruginosa* in Switzerland 2022–2023. Eur. J. Clin. Microbiol. Infect. Dis..

[46] Uskudar-Guclu A., Danyildiz S., Mirza H.C., Ok M.A., Basustaoglu A. (2024). In vitro activity of cefiderocol against carbapenem-resistant Acinetobacter baumannii carrying various β-lactamase encoding genes. Eur. J. Clin. Microbiol. Infect. Dis..

[47] Bulens S.N., Campbell D., McKay S.L., Vlachos N., Burgin A., Burroughs M., Padila J., Grass J.E., Jacob J.T., Smith G. (2024). Carbapenem-resistant Acinetobacter baumannii complex in the United States—An epidemiological and molecular description of isolates collected through the Emerging Infections Program, 2019. Am. J. Infect. Control..

[48] Kayama S., Kawakami S., Kondo K., Kitamura N., Yu L., Hayashi W., Yahara K., Sugawara Y., Sugai M. (2024). In vitro activity of cefiderocol against carbapenemase-producing and meropenem-non-susceptible Gram-negative bacteria collected in the Japan Antimicrobial Resistant Bacterial Surveillance. J. Glob. Antimicrob. Resist..

[49] Dahdouh E., Gómez-Marcos L., Cañada-García J.E., de Arellano E.R., Sánchez-García A., Sánchez-Romero I., López-Urrutia L., de la Iglesia P., Gonzalez-Praetorius A., Sotelo J. (2024). Characterizing carbapenemase-producing *Escherichia coli* isolates from Spain: High genetic heterogeneity and wide geographical spread. Front. Cell. Infect. Microbiol..

[50] Ito A., Sato T., Ota M., Takemura M., Nishikawa T., Toba S., Kohira N., Miyagawa S., Ishibashi N., Matsumoto S. (2017). In Vitro Antibacterial Properties of Cefiderocol, a Novel Siderophore Cephalosporin, against Gram-Negative Bacteria. Antimicrob. Agents Chemother..

[51] Luscher A., Moynié L., Auguste P.S., Bumann D., Mazza L., Pletzer D., Naismith J.H., Köhler T. (2018). TonB-Dependent Receptor Repertoire of Pseudomonas aeruginosa for Uptake of Siderophore-Drug Conjugates. Antimicrob. Agents Chemother..

[52] Gomis-Font M.A., Clari M.A., López-Causapé C., Navarro D., Oliver A. (2024). Emergence of cefiderocol resistance during ceftazidime/avibactam treatment caused by a large genomic deletion, including *ampD* and *piuCD* genes, in *Pseudomonas aeruginosa*. Antimicrob. Agents Chemother..

[53] Streling A.P., Al Obaidi M.M., Lainhart W.D., Zangeneh T., Khan A., Dinh A.Q., Hanson B., Arias C.A., Miller W.R. (2021). Evolution of Cefiderocol Non-Susceptibility in *Pseudomonas aeruginosa* in a Patient Without Previous Exposure to the Antibiotic. Clin. Infect. Dis..

[54] Malik S., Kaminski M., Landman D., Quale J. (2020). Cefiderocol Resistance in *Acinetobacter baumannii*: Roles of β-Lactamases, Siderophore Receptors, and Penicillin Binding Protein 3. Antimicrob. Agents Chemother..

[55] Yamano Y., Ishibashi N., Kuroiwa M., Takemura M., Sheng W.-H., Hsueh P.-R. (2021). Characterisation of cefiderocol-non-susceptible Acinetobacter baumannii isolates from Taiwan. J. Glob. Antimicrob. Resist..

[56] Findlay J., Bianco G., Boattini M., Nordmann P. (2024). In vivo development of cefiderocol resistance in carbapenem-resistant *Acinetobacter baumannii* associated with the downregulation of a TonB-dependent siderophore receptor, PiuA. J. Antimicrob. Chemother..

[57] Huang E., Thompson R.N., Moon S.H., Keck J.M., Lowry M.S., Melero J., Jun S.-R., Rosenbaum E.R., Dare R.K. (2024). Treatment-emergent cefiderocol resistance in carbapenem-resistant *Acinetobacter baumannii* is associated with insertion sequence IS *Aba36* in the siderophore receptor *pirA*. Antimicrob. Agents Chemother..

[58] Nurjadi D., Kocer K., Chanthalangsy Q., Klein S., Heeg K., Boutin S. (2022). New Delhi Metallo-Beta-Lactamase Facilitates the Emergence of Cefiderocol Resistance in Enterobacter cloacae. Antimicrob. Agents Chemother..

[59] McElheny C.L., Fowler E.L., Iovleva A., Shields R.K., Doi Y. (2021). In Vitro Evolution of Cefiderocol Resistance in an NDM-Producing Klebsiella pneumoniae Due to Functional Loss of CirA. Microbiol. Spectr..

[60] Padovani M., Bertelli A., Corbellini S., Piccinelli G., Gurrieri F., De Francesco M.A. (2023). In Vitro Activity of Cefiderocol on Multiresistant Bacterial Strains and Genomic Analysis of Two Cefiderocol Resistant Strains. Antibiotics.

[61] Bao J., Xie L., Ma Y., An R., Gu B., Wang C. (2022). Proteomic and Transcriptomic Analyses Indicate Reduced Biofilm-Forming Abilities in Cefiderocol-Resistant Klebsiella pneumoniae. Front. Microbiol..

[62] Sato T., Ito A., Ishioka Y., Matsumoto S., Rokushima M., Kazmierczak K.M., Hackel M., Sahm D.F., Yamano Y. (2020). Escherichia coli strains possessing a four amino acid YRIN insertion in PBP3 identified as part of the SIDE-RO-WT-2014 surveillance study. JAC-Antimicrob. Resist..

[63] Price T.K., Davar K., Contreras D., Ward K.W., Garner O.B., Simner P.J., Yang S., Chandrasekaran S. (2021). Case Report and Genomic Analysis of Cefiderocol-Resistant *Escherichia coli* Clinical Isolates. Am. J. Clin. Pathol..

[64] Lan P., Lu Y., Chen Z., Wu X., Hua X., Jiang Y., Zhou J., Yu Y. (2022). Emergence of High-Level Cefiderocol Resistance in Carbapenem-Resistant *Klebsiella pneumoniae* from Bloodstream Infections in Patients with Hematologic Malignancies in China. Microbiol. Spectr..

[65] Tascini C., Coppi M., Antonelli A., Niccolai C., Bartolini A., Pecori D., Sartor A., Giani T., Rossolini G.M. (2024). In vivo evolution to high-level cefiderocol resistance of NDM-1–producing *Klebsiella pneumoniae*, followed by intra-hospital cross-transmission. Clin. Microbiol. Infect..

[66] Barker K.R., Rebick G.W., Fakharuddin K., MacDonald C., Mulvey M.R., Mataseje L.F. (2024). When the Trojan horse is unable to reach inside the city: Investigation of the mechanism of resistance behind the first reported cefiderocol-resistant *E. coli* in Canada. Microbiol. Spectr..

[67] Arcari G., Cecilia F., Oliva A., Polani R., Raponi G., Sacco F., De Francesco A., Pugliese F., Carattoli A. (2023). Genotypic Evolution of *Klebsiella pneumoniae* Sequence Type 512 during Ceftazidime/Avibactam, Meropenem/Vaborbactam, and Cefiderocol Treatment, Italy. Emerg. Infect. Dis..

[68] Gill C.M., Abdelraouf K., Oota M., Nakamura R., Kuroiwa M., Gahara Y., Takemura M., Yamano Y., Nicolau D.P. (2021). Discrepancy in sustained efficacy and resistance emergence under human-simulated exposure of cefiderocol against *Stenotrophomonas maltophilia* between *in vitro* chemostat and *in vivo* murine infection models. J. Antimicrob. Chemother..

[69] Werth B.J., Ashford N.K., Penewit K., Waalkes A., Holmes E.A., Bryan A., Salipante S.J. (2022). Evolution of cefiderocol resistance in *Stenotrophomonas maltophilia* using *in vitro* serial passage techniques. JAC-Antimicrob. Resist..

[70] Kohira N., Hackel M.A., Ishioka Y., Kuroiwa M., Sahm D.F., Sato T., Maki H., Yamano Y. (2020). Reduced susceptibility mechanism to cefiderocol, a siderophore cephalosporin, among clinical isolates from a global surveillance programme (SIDERO-WT-2014). J. Glob. Antimicrob. Resist..

[71] Poirel L., Sadek M., Nordmann P. (2021). Contribution of PER-Type and NDM-Type β-Lactamases to Cefiderocol Resistance in *Acinetobacter baumannii*. Antimicrob. Agents Chemother..

[72] Poirel L., de la Rosa J.-M.O., Sadek M., Nordmann P. (2022). Impact of Acquired Broad-Spectrum β-Lactamases on Susceptibility to Cefiderocol and Newly Developed β-Lactam/β-Lactamase Inhibitor Combinations in *Escherichia coli* and *Pseudomonas aeruginosa*. Antimicrob. Agents Chemother..

[73] Fröhlich C., Sørum V., Tokuriki N., Johnsen P.J., Samuelsen Ø. (2022). Evolution of β-lactamase-mediated cefiderocol resistance. J. Antimicrob. Chemother..

[74] Mezcord V., Traglia G.M., Pasteran F., Escalante J., Lopez C., Wong O., Rojas L., Marshall S.H., Tolmasky M.E., Bonomo R.A. (2024). Characterisation of cefiderocol-resistant spontaneous mutant variants of *Klebsiella pneumoniae*-producing NDM-5 with a single mutation in cirA. Int. J. Antimicrob. Agents.

[75] Simner P.J., Mostafa H.H., Bergman Y., Ante M., Tekle T., Adebayo A., Beisken S., Dzintars K., Tamma P.D. (2021). Progressive Development of Cefiderocol Resistance in *Escherichia coli* During Therapy is Associated with an Increase in *bla*NDM-5 Copy Number and Gene Expression. Clin. Infect. Dis..

[76] Hobson C.A., Cointe A., Jacquier H., Choudhury A., Magnan M., Courroux C., Tenaillon O., Bonacorsi S., Birgy A. (2021). Cross-resistance to cefiderocol and ceftazidime–avibactam in KPC β-lactamase mutants and the inoculum effect. Clin. Microbiol. Infect..

[77] Poirel L., Sadek M., Kusaksizoglu A., Nordmann P. (2022). Co-resistance to ceftazidime-avibactam and cefiderocol in clinical isolates producing KPC variants. Eur. J. Clin. Microbiol. Infect. Dis..

[78] Bianco G., Boattini M., Comini S., Iannaccone M., Bondi A., Cavallo R., Costa C. (2022). In vitro activity of cefiderocol against ceftazidime-avibactam susceptible and resistant KPC-producing Enterobacterales: Cross-resistance and synergistic effects. Eur. J. Clin. Microbiol. Infect. Dis..

[79] Gaibani P., Amadesi S., Lazzarotto T., Ambretti S. (2022). Genome characterization of a Klebsiella pneumoniae co-producing OXA-181 and KPC-121 resistant to ceftazidime/avibactam, meropenem/vaborbactam, imipenem/relebactam and cefiderocol isolated from a critically ill patient. J. Glob. Antimicrob. Resist..

[80] Di Pilato V., Codda G., Niccolai C., Willison E., Wong J.L., Coppo E., Frankel G., Marchese A., Rossolini G.M. (2024). Functional features of KPC-109, a novel 270-loop KPC-3 mutant mediating resistance to avibactam-based β-lactamase inhibitor combinations and cefiderocol. Int. J. Antimicrob. Agents.

[81] Amadesi S., Bianco G., Secci B., Fasciana T., Boattini M., Costa C., Gaibani P. (2024). Complete Genome Sequence of a *Klebsiella pneumoniae* Strain Carrying Novel Variant *bla*_KPC-203_, Cross-Resistant to Ceftazidime/Avibactam and Cefiderocol, but Susceptible to Carbapenems, Isolated in Italy, 2023. Pathogens.

[82] Giufrè M., Errico G., Del Grosso M., Pagnotta M., Palazzotti B., Ballardini M., Pantosti A., Meledandri M., Monaco M. (2024). Detection of KPC-216, a Novel KPC-3 Variant, in a Clinical Isolate of *Klebsiella pneumoniae* ST101 Co-Resistant to Ceftazidime-Avibactam and Cefiderocol. Antibiotics.

[83] Jacob A.-S., Chong G.-L., Lagrou K., Depypere M., Desmet S. (2021). No in vitro activity of cefiderocol against OXA-427-producing Enterobacterales. J. Antimicrob. Chemother..

[84] Shields R.K., Iovleva A., Kline E.G., Kawai A., McElheny C.L., Doi Y. (2020). Clinical Evolution of AmpC-Mediated Ceftazidime-Avibactam and Cefiderocol Resistance in *Enterobacter cloacae* Complex Following Exposure to Cefepime. Clin. Infect. Dis..

[85] Nordmann P., Shields R.K., Doi Y., Takemura M., Echols R., Matsunaga Y., Yamano Y. (2022). Mechanisms of Reduced Susceptibility to Cefiderocol Among Isolates from the CREDIBLE-CR and APEKS-NP Clinical Trials. Microb. Drug Resist..

[86] Kawai A., Shropshire W.C., Suzuki M., Borjan J., Aitken S.L., Bachman W.C., McElheny C.L., Bhatti M.M., Shields R.K., Shelburne S.A. (2024). Structural insights into the molecular mechanism of high-level ceftazidime–avibactam resistance conferred by CMY-185. mBio.

[87] Amadesi S., Gatti M., Rinaldi M., Pea F., Viale P., Gaibani P. (2024). Novel CMY-186 variant conferring cross-resistance to cefiderocol and ceftazidime/avibactam in a Klebsiella pneumoniae from a critically ill patient during cefiderocol and ceftazidime/avibactam treatments. Int. J. Antimicrob. Agents.

[88] Simner P.J., Beisken S., Bergman Y., Posch A.E., Cosgrove S.E., Tamma P.D. (2021). Cefiderocol Activity Against Clinical Pseudomonas aeruginosa Isolates Exhibiting Ceftolozane-Tazobactam Resistance. Open Forum Infect. Dis..

[89] Ballesté-Delpierre C., Ramírez Á., Muñoz L., Longshaw C., Roca I., Vila J. (2022). Assessment of In Vitro Cefiderocol Susceptibility and Comparators against an Epidemiologically Diverse Collection of *Acinetobacter baumannii* Clinical Isolates. Antibiotics.

[90] Liu C., Yi J., Lu M., Yang P., Du C., Jiang F., Du P., Shen N. (2024). Dynamic within-host cefiderocol heteroresistance caused by blaSHV-12 amplification in pandrug-resistant and hypervirulent Klebsiella pneumoniae sequence type 11. Drug Resist. Updat..

[91] Rolston K.V.I., Gerges B., Shelburne S., Aitken S.L., Raad I., Prince R.A. (2020). Activity of Cefiderocol and Comparators against Isolates from Cancer Patients. Antimicrob. Agents Chemother..

[92] Simner P.J., Beisken S., Bergman Y., Ante M., Posch A.E., Tamma P.D. (2022). Defining Baseline Mechanisms of Cefiderocol Resistancein the Enterobacterales. Microb. Drug Resist..

[93] Ito A., Nishikawa T., Ishii R., Kuroiwa M., Ishioka Y., Kurihara N., Sakikawa I., Ota T., Rokushima M., Tsuji M. (2018). 696. Mechanism of Cefiderocol high MIC mutants obtained in non-clinical FoR studies. Open Forum Infect. Dis..

[94] Magallon A., Amoureux L., Garrigos T., Sonois M., Varin V., Neuwirth C., Bador J. (2022). Role of AxyABM overexpression in acquired resistance in *Achromobacter xylosoxidans*. J. Antimicrob. Chemother..

[95] Yamano Y., Nakamura R., Takemura M., Echols R. (2020). 1455. Potential Mechanisms of Cefiderocol MIC Increase in Enterobacterales in *In Vitro* Resistance Acquisition Studies. Open Forum Infect. Dis. Oxf. Acad..

[96] Zhang Q., Neidig N., Chu T.-Y., Divoky C., Carpenter J., Lee-Hsiao C., Threatt H., Sultana R., Bush K. (2022). In vitro antibacterial activity of cefiderocol against recent multidrug-resistant carbapenem-nonsusceptible Enterobacterales isolates. Diagn. Microbiol. Infect. Dis..

[97] Bianco G., Gaibani P., Comini S., Boattini M., Banche G., Costa C., Cavallo R., Nordmann P. (2022). Synergistic Effect of Clinically Available Beta-Lactamase Inhibitors Combined with Cefiderocol against Carbapenemase-Producing Gram-Negative Organisms. Antibiotics.

[98] Bilal M., El Tabei L., Büsker S., Krauss C., Fuhr U., Taubert M. (2021). Clinical Pharmacokinetics and Pharmacodynamics of Cefiderocol. Clin. Pharmacokinet..

[99] Katsube T., Echols R., Wajima T. (2019). Pharmacokinetic and Pharmacodynamic Profiles of Cefiderocol, a Novel Siderophore Cephalosporin. Clin. Infect. Dis..

[100] Chen I.H., Kidd J.M., Abdelraouf K., Nicolau D.P. (2019). Comparative *In Vivo* Antibacterial Activity of Human-Simulated Exposures of Cefiderocol and Ceftazidime against *Stenotrophomonas maltophilia* in the Murine Thigh Model. Antimicrob. Agents Chemother..

[101] Monogue M.L., Tsuji M., Yamano Y., Echols R., Nicolau D.P. (2017). Efficacy of Humanized Exposures of Cefiderocol (S-649266) against a Diverse Population of Gram-Negative Bacteria in a Murine Thigh Infection Model. Antimicrob. Agents Chemother..

[102] Ghazi I.M., Monogue M.L., Tsuji M., Nicolau D.P. (2018). Pharmacodynamics of cefiderocol, a novel siderophore cephalosporin, in a Pseudomonas aeruginosa neutropenic murine thigh model. Int. J. Antimicrob. Agents.

[103] Stainton S.M., Monogue M.L., Tsuji M., Yamano Y., Echols R., Nicolau D.P., Stainton S.M., Monogue M.L., Tsuji M., Yamano Y. (2019). Efficacy of Humanized Cefiderocol Exposures over 72 Hours against a Diverse Group of Gram-Negative Isolates in the Neutropenic Murine Thigh Infection Model. Antimicrob. Agents Chemother..

[104] Nakamura R., Ito-Horiyama T., Takemura M., Toba S., Matsumoto S., Ikehara T., Tsuji M., Sato T., Yamano Y. (2019). In Vivo Pharmacodynamic Study of Cefiderocol, a Novel Parenteral Siderophore Cephalosporin, in Murine Thigh and Lung Infection Models. Antimicrob. Agents Chemother..

[105] Sumi C.D., Heffernan A.J., Lipman J., Roberts J.A., Sime F.B. (2019). What Antibiotic Exposures Are Required to Suppress the Emergence of Resistance for Gram-Negative Bacteria? A Systematic Review. Clin. Pharmacokinet..

[106] Gatti M., Cojutti P.G., Pea F. (2024). Impact of attaining aggressive vs. conservative PK/PD target on the clinical efficacy of beta-lactams for the treatment of Gram-negative infections in the critically ill patients: A systematic review and meta-analysis. Crit. Care.

[107] Gatti M., Bartoletti M., Cojutti P.G., Gaibani P., Conti M., Giannella M., Viale P., Pea F. (2021). A descriptive case series of pharmacokinetic/pharmacodynamic target attainment and microbiological outcome in critically ill patients with documented severe extensively drug-resistant Acinetobacter baumannii bloodstream infection and/or ventilator-associated pneumonia treated with cefiderocol. J. Glob. Antimicrob. Resist..

[108] Pinna S.M., Corcione S., De Nicolò A., Montrucchio G., Scabini S., Vita D., De Benedetto I., Lupia T., Mula J., Di Perri G. (2022). Pharmacokinetic of Cefiderocol in Critically Ill Patients Receiving Renal Replacement Therapy: A Case Series. Antibiotics.

[109] Gatti M., Giannella M., Rinaldi M., Gaibani P., Viale P., Pea F. (2022). Pharmacokinetic/Pharmacodynamic Analysis of Continuous-Infusion Fosfomycin in Combination with Extended-Infusion Cefiderocol or Continuous-Infusion Ceftazidime-Avibactam in a Case Series of Difficult-to-Treat Resistant *Pseudomonas aeruginosa* Bloodstream Infections and/or Hospital-Acquired Pneumonia. Antibiotics.

[110] Gatti M., Rinaldi M., Tonetti T., Gaibani P., Siniscalchi A., Viale P., Pea F. (2023). Pharmacokinetics/pharmacodynamics of cefiderocol administered by continuous infusion in a case series of critically ill patients with carbapenem-resistant Acinetobacter baumannii infections undergoing continuous venovenous haemodiafiltration (CVVHDF). Int. J. Antimicrob. Agents.

[111] Saravolatz L.D., Pea F., Viale P. (2006). The Antimicrobial Therapy Puzzle: Could Pharmacokinetic-Pharmacodynamic Relationships Be Helpful in Addressing the Issue of Appropriate Pneumonia Treatment in Critically Ill Patients?. Clin. Infect. Dis..

[112] Katsube T., Nicolau D.P., Rodvold K.A., Wunderink R.G., Echols R., Matsunaga Y., Menon A., Portsmouth S., Wajima T. (2021). Intrapulmonary pharmacokinetic profile of cefiderocol in mechanically ventilated patients with pneumonia. J. Antimicrob. Chemother..

[113] Kawaguchi N., Katsube T., Echols R., Wajima T., Nicolau D.P. (2022). Intrapulmonary Pharmacokinetic Modeling and Simulation of Cefiderocol, a Parenteral Siderophore Cephalosporin, in Patients with Pneumonia and Healthy Subjects. J. Clin. Pharmacol..

[114] Sacks D., Baxter B., Campbell B.C.V., Carpenter J.S., Cognard C., Dippel D., Eesa M., Fischer U., Hausegger K., Hirsch J.A. (2018). Multisociety Consensus Quality Improvement Revised Consensus Statement for Endovascular Therapy of Acute Ischemic Stroke. Int. J. Stroke.

[115] Möhlmann J.E., van Luin M., Uijtendaal E.V., Zahr N., Sikma M.A. (2023). Continuous infusion of cefiderocol in a critically ill patient with continuous venovenous haemofiltration. Br. J. Clin. Pharmacol..

[116] Gatti M., Pea F. (2023). Jumping into the future: Overcoming pharmacokinetic/pharmacodynamic hurdles to optimize the treatment of severe difficult to treat-Gram-negative infections with novel beta-lactams. Expert Rev. Anti-Infect. Ther..

[117] Naseer S., Weinstein E.A., Rubin D.B., Suvarna K., Wei X., Higgins K., Goodwin A., Jang S.H., Iarikov D., Farley J. (2021). US Food and Drug Administration (FDA): Benefit-Risk Considerations for Cefiderocol (Fetroja®). Clin. Infect. Dis..

[118] Portsmouth S., van Veenhuyzen D., Echols R., Machida M., Ferreira J.C.A., Ariyasu M., Tenke P., Den Nagata T. (2017). Cefiderocol compared with imipenem/cirastatin in the treatment of adults with complicated urinary tract infections with or without pyelonephritis or acute uncomplicated pyelonephritis: Results from a multicenter, double-blind, randomized study. Eur. Congr. Clin. Microbiol. Infect. Dis..

[119] EMA (2020). Fetcroja. European Medicines Agency. https://www.ema.europa.eu/en/medicines/human/EPAR/fetcroja.

[120] de la Fuente C., Rodríguez M., Merino N., Carmona P., Machuca I., Córdoba-Fernández M., Guzmán-Puche J., Domínguez A., López-Viñau T., García L. (2023). Real-life use of cefiderocol for salvage therapy of severe infections due to carbapenem-resistant Gram-negative bacteria. Int. J. Antimicrob. Agents.

[121] Gavaghan V., Miller J.L., Dela-Pena J. (2023). Case series of cefiderocol for salvage therapy in carbapenem-resistant Gram-negative infections. Infection.

[122] Vacheron C.-H., Kaas A., Rasigade J.-P., Aubrun F., Argaud L., Balanca B., Fellahi J.-L., Richard J.C., Lukaszewicz A.-C., Wallet F. (2024). Correction: Cefiderocol in Difficult-to-Treat Nf-GNB in ICU Settings. Ann. Intensiv. Care.

[123] Cai B., Nguyen S.T., Copeland J.D., Song H.J., Slover C.M. (2023). 2751. Cefiderocol Use in Treating Patients with Confirmed *Stenotrophomonas maltophilia* Infections in US Hospitals During January 2020–June 2022. Open Forum Infect. Dis..

[124] Timsit J.F., Paul M., Shields R.K., Echols R., Baba T., Yamano Y., Portsmouth S. (2022). Cefiderocol for the Treatment of Infections Due to Metallo-B-lactamase–Producing Pathogens in the CREDIBLE-CR and APEKS-NP Phase 3 Randomized Studies. Clin. Infect. Dis..

[125] Falcone M., Tiseo G. (2022). Cefiderocol for the Treatment of Metallo-β-Lactamases Producing Gram-Negative Bacilli: Lights and Shadows From the Literature. Clin. Infect. Dis..

[126] Bavaro D.F., Belati A., Diella L., Stufano M., Romanelli F., Scalone L., Stolfa S., Ronga L., Maurmo L., Dell’aera M. (2021). Cefiderocol-Based Combination Therapy for “Difficult-to-Treat” Gram-Negative Severe Infections: Real-Life Case Series and Future Perspectives. Antibiotics.

[127] Falcone M., Tiseo G., Nicastro M., Leonildi A., Vecchione A., Casella C., Forfori F., Malacarne P., Guarracino F., Barnini S. (2021). Cefiderocol as Rescue Therapy for Acinetobacter baumannii and Other Carbapenem-resistant Gram-negative Infections in Intensive Care Unit Patients. Clin. Infect. Dis..

[128] Bavaro D.F., Papagni R., Belati A., Diella L., De Luca A., Brindicci G., De Gennaro N., Di Gennaro F., Romanelli F., Stolfa S. (2023). Cefiderocol Versus Colistin for the Treatment of Carbapenem-Resistant *Acinetobacter baumannii* Complex Bloodstream Infections: A Retrospective, Propensity-Score Adjusted, Monocentric Cohort Study. Infect. Dis. Ther..

[129] Dalfino L., Stufano M., Bavaro D.F., Diella L., Belati A., Stolfa S., Romanelli F., Ronga L., Di Mussi R., Murgolo F. (2023). Effectiveness of First-Line Therapy with Old and Novel Antibiotics in Ventilator-Associated Pneumonia Caused by Carbapenem-Resistant *Acinetobacter baumannii*: A Real Life, Prospective, Observational, Single-Center Study. Antibiotics.

[130] Rando E., Cutuli S.L., Sangiorgi F., Tanzarella E.S., Giovannenze F., De Angelis G., Murri R., Antonelli M., Fantoni M., De Pascale G. (2023). Cefiderocol-containing regimens for the treatment of carbapenem-resistant *A. baumannii* ventilator-associated pneumonia: A propensity-weighted cohort study. JAC-Antimicrob. Resist..

[131] Russo A., Bruni A., Gullì S., Borrazzo C., Quirino A., Lionello R., Serapide F., Garofalo E., Serraino R., Romeo F. (2023). Efficacy of cefiderocol- vs colistin-containing regimen for treatment of bacteraemic ventilator-associated pneumonia caused by carbapenem-resistant *Acinetobacter baumannii* in patients with COVID-19. Int. J. Antimicrob. Agents.

[132] Oliva A., Liguori L., Covino S., Petrucci F., Cogliati-Dezza F., Curtolo A., Savelloni G., Comi M., Sacco F., Ceccarelli G. (2024). Clinical effectiveness of cefiderocol for the treatment of bloodstream infections due to carbapenem-resistant *Acinetobacter baumannii* during the COVID-19 era: A single center, observational study. Eur. J. Clin. Microbiol. Infect. Dis..

[133] Pascale R., Pasquini Z., Bartoletti M., Caiazzo L., Fornaro G., Bussini L., Volpato F., Marchionni E., Rinaldi M., Trapani F. (2021). Cefiderocol treatment for carbapenem-resistant *Acinetobacter baumannii* infection in the ICU during the COVID-19 pandemic: A multicentre cohort study. JAC-Antimicrob. Resist..

[134] Giannella M., Verardi S., Karas A., Hadi H.A., Dupont H., Soriano A., Henriksen A.S., Cooper A., Falcone M., ARES Study Group (2023). Carbapenem-Resistant *Acinetobacter* spp. Infection in Critically Ill Patients With Limited Treatment Options: A Descriptive Study of Cefiderocol Therapy During the COVID-19 Pandemic. Open Forum Infect. Dis..

[135] World Health Organization Regional Office for Europe, European Centre for Disease Prevention and Control (2022). Antimicrobial Resistance Surveillance Europe. https://www.ecdc.europa.eu/sites/default/files/documents/Joint-WHO-ECDC-AMR-report-2022.pdf.

[136] Satlin M.J., Simner P.J., Slover C.M., Yamano Y., Nagata T.D., Portsmouth S. (2023). Cefiderocol Treatment for Patients with Multidrug- and Carbapenem-Resistant *Pseudomonas aeruginosa* Infections in the Compassionate Use Program. Antimicrob. Agents Chemother..

[137] Ramirez P. Real-world effectiveness and safety of cefiderocol in patients with Gram-negative bacterial in-fections in the early access programme in Spain: Results of the PERSEUS study. Abstract. ECCMID.

[138] Paez J.G., Costa S. (2008). Risk factors associated with mortality of infections caused by *Stenotrophomonas maltophilia*: A systematic review. J. Hosp. Infect..

[139] Isler B., Vatansever C., Özer B., Çınar G., Aslan A.T., Falconer C., Bauer M.J., Forde B., Şimşek F., Tülek N. (2023). Higher rates of cefiderocol resistance among NDM producing Klebsiella bloodstream isolates applying EUCAST over CLSI breakpoints. Infect. Dis..

[140] Tamma P.D., Heil E.L., Justo J.A., Mathers A.J., Satlin M.J., Bonomo R.A. Infectious Diseases Society of America Antimicrobial-Resistant Treatment Guidance: Gram-Negative Bacterial Infections. Infectious Diseases Society of America 2024; Version 4.0. https://www.idsociety.org/practice-guideline/amr-guidance/.

[141] Marino A., Stracquadanio S., Campanella E., Munafò A., Gussio M., Ceccarelli M., Bernardini R., Nunnari G., Cacopardo B. (2023). Intravenous Fosfomycin: A Potential Good Partner for Cefiderocol. Clinical Experience and Considerations. Antibiotics.

[142] Paranos P., Vourli S., Pournaras S., Meletiadis J. (2022). Assessing Clinical Potential of Old Antibiotics against Severe Infections by Multi-Drug-Resistant Gram-Negative Bacteria Using In Silico Modelling. Pharmaceuticals.

[143] Boattini M., Bianco G., Comini S., Costa C., Gaibani P. (2024). In vivo development of resistance to novel β-lactam/β-lactamase inhibitor combinations in KPC-producing *Klebsiella pneumoniae* infections: A case series. Eur. J. Clin. Microbiol. Infect. Dis..

[144] Mezcord V., Escalante J., Nishimura B., Traglia G.M., Sharma R., Vallé Q., Tuttobene M.R., Subils T., Marin I., Pasteran F. (2023). Induced Heteroresistance in Carbapenem-Resistant *Acinetobacter baumannii* (CRAB) via Exposure to Human Pleural Fluid (HPF) and Its Impact on Cefiderocol Susceptibility. Int. J. Mol. Sci..

[145] Amadesi S., Amedeo A., Rinaldi M., Palombo M., Giannella M., Gaibani P. (2024). In vivo emergence of cefiderocol and ceftazidime/avibactam cross-resistance in KPC-producing Klebsiella pneumoniae following ceftazidime/avibactam -based therapies. Diagn. Microbiol. Infect. Dis..

[146] Bovo F., Amadesi S., Palombo M., Lazzarotto T., Ambretti S., Gaibani P. (2023). Clonal dissemination of *Klebsiella pneumoniae* resistant to cefiderocol, ceftazidime/avibactam, meropenem/vaborbactam and imipenem/relebactam co-producing KPC and OXA-181 carbapenemase. JAC-Antimicrob. Resist..

[147] Lewis R.E., Palombo M., Diani E., Secci B., Gibellini D., Gaibani P. (2024). Synergistic Activity of Cefiderocol in Combination with Avibactam, Sulbactam or Tazobactam against Carbapenem-Resistant Gram-Negative Bacteria. Cells.

